# The Interplay of Glucagon-Like Peptide-1 Receptor Trafficking and Signalling in Pancreatic Beta Cells

**DOI:** 10.3389/fendo.2021.678055

**Published:** 2021-05-10

**Authors:** Amaara Marzook, Alejandra Tomas, Ben Jones

**Affiliations:** ^1^ Section of Endocrinology and Investigative Medicine, Imperial College London, London, United Kingdom; ^2^ Section of Cell Biology and Functional Genomics, Imperial College London, London, United Kingdom

**Keywords:** GLP-1, GLP-1R, biased agonism, receptor trafficking, pancreatic beta cells

## Abstract

The glucagon-like peptide 1 receptor (GLP-1R) is a class B G protein-coupled receptor (GPCR) which mediates the effects of GLP-1, an incretin hormone secreted primarily from L-cells in the intestine and within the central nervous system. The GLP-1R, upon activation, exerts several metabolic effects including the release of insulin and suppression of appetite, and has, accordingly, become an important target for the treatment for type 2 diabetes (T2D). Recently, there has been heightened interest in how the activated GLP-1R is trafficked between different endomembrane compartments, controlling the spatial origin and duration of intracellular signals. The discovery of “biased” GLP-1R agonists that show altered trafficking profiles and selective engagement with different intracellular effectors has added to the tools available to study the mechanisms and physiological importance of these processes. In this review we survey early and recent work that has shed light on the interplay between GLP-1R signalling and trafficking, and how it might be therapeutically tractable for T2D and related diseases.

## Introduction

Recent estimates suggest a global prevalence of approximately 422 million people living with diabetes, a number expected to rise to 629 million by 2045 (1). Type 2 diabetes (T2D), the commonest form, arises due to a combination of genetic and lifestyle factors that contribute to defective production of insulin from pancreatic beta cells and resistance to its action in peripheral and central tissues (2). Prolonged elevation of blood glucose, as well as related adverse metabolic features such as dyslipidaemia and chronic inflammation, lead ultimately to a range of serious health consequences, including cardiovascular disease, limb amputation, blindness, and kidney failure. Existing anti-diabetic drugs contribute to improve glycaemic control by either increasing the body’s sensitivity to insulin, enhancing insulin secretion or reducing renal glucose reabsorption. However, these drugs are reported to cause a myriad of adverse effects such as weight gain, oedema, intestinal discomfort and hypoglycaemia (3).

Glucagon-like peptide 1 receptor agonists (GLP-1RAs) have emerged as safe and effective treatments for diabetes (4). The GLP-1R, once activated, initiates effects that lead to an overall decrease in blood glucose levels, including potentiation of insulin secretion, reduced glucagon secretion, and weight loss *via* satiety induction that results in increased insulin sensitivity (5). GLP-1RAs work to stimulate insulin secretion in a glucose-dependent manner, therefore carrying a low risk of hypoglycaemia. To date, they represent the only G protein-coupled receptor (GPCR) ligands approved as glucose-lowering agents.

Once activated, GLP-1Rs are transported by the cellular endocytic machinery to different subcellular compartments, allowing qualitative and quantitative fine-tuning of intracellular signalling responses linked to insulin secretion and other downstream effects (6). Understanding these complex trafficking mechanisms could potentially provide an avenue for further development of compounds with optimised therapeutic characteristics. In particular, there has been substantial recent interest in agonists that have a high selectivity for particular signalling effectors or pathways, a concept also known as “biased agonism” (7). This phenomenon is strongly associated with alterations to GLP-1R trafficking, although the causal relationships are not fully understood. In this review we focus on the role of GLP-1R trafficking as a critical component of GLP-1R agonism, and how it may be possible to harness some of these processes to improve therapeutic targeting of the GLP-1R in T2D.

## GLP-1R Signalling in Pancreatic Beta Cells

The GLP-1R is a class B (secretin family) GPCR. Activation of the GLP-1R in pancreatic beta cells results in insulin synthesis, potentiation of glucose-stimulated insulin secretion (GSIS), and longer-term effects on beta cell survival, proliferation and neogenesis. An early study used RNA nuclease protection combined with Southern blotting of reverse-transcribed PCR products to map *Glp1r* in rat tissues, identifying several sources of expression including pancreas, lung, kidney, stomach, intestine and brain (8). *GLP1R* mRNA transcripts were also reported in the human retina and retinas of *db/db* mice (9), and in immune cells such as invariant natural killer T (iKNT) cells (10). Historically, immunohistochemical confirmation has been hampered by the poor specificity of anti-GLP-1R antibodies (11), but better performing antibodies have enabled the distribution of GLP-1R to be more confidently ascertained in rodents, monkeys and humans (12–14). Certain species differences are apparent from these studies, with much higher levels of GLP-1R in rodent compared to human lung tissue, and absent expression in human thyroid. Comparisons of mRNA *versus* protein distribution typically deliver congruent results, with exceptions including GLP-1R protein detected on neuronal projections distant from the their respective *Glp1r* transcript-containing cell bodies (14), and instances where low abundance *Glp1r* transcripts, e.g. in pancreatic delta cells, do not yield detectable protein expression (15). Reporter mice, in which active *Glp1r* promoter-dependent Cre recombinase leads to fluorescent protein expression, have also been used to map GLP-1R-containing cell types (16–19). The most recent study by Andersen et al. (19) revealed additional GLP-1R-expressing cell types such as tracheal cartilage chondrocytes and skin fibroblasts that had not been identified with the original *Glp1r* reporter mouse (16); it is not known if these observations are applicable to humans. A further approach involves the use of radiolabelled (20) or, more recently, fluorescent peptides (21) to define GLP-1R ligand binding sites, revealing for example sub-populations of GLP-1R-expressing pancreatic alpha cells.

Human GLP-1R comprises 463 amino acids, including an N-terminal signal peptide that is cleaved on delivery to the plasma membrane (22). GLP-1R and related class B GPCRs are characterised by a stereotyped extracellular N-terminal domain crucial for ligand recognition, an intracellular C-terminus, and seven transmembrane (7TM) α-helices spanning the plasma membrane that are connected by three intracellular loops (ICL1-ICL3) and three extracellular loops (ECL1-ECL3) on either side of the membrane. Class B GPCRs typically bind their peptide ligands through a mechanism known as the two-domain model, in which the extracellular domain (ECD) binds to the C-terminal end of the ligand first, enabling a second interaction between the N-terminus of the ligand and the 7TM domains of the receptor (22). Recent advances in GPCR structural biology have provided insights into GLP-1R-ligand interactions that underpin binding and activation processes (23–26).

### Gα_s_/cAMP/PKA Signalling in Beta Cells

Canonical GLP-1R signalling occurs *via* heterotrimeric G proteins, comprising an independent Gα subunit and a Gβ/γ dimer bound reversibly to Gα. The Gα subunits can be classified into 4 subtypes: Gα_i_, Gα_0_, Gα_s_, and Gα_q/11_, based on the nature of their interaction with specific downstream effectors. Agonist-activated GPCRs act as guanyl nucleotide exchange factors (GEFs) for G proteins. The nucleotide binding pocket opens, allowing exchange of inactive guanine diphosphate (GDP) for guanine triphosphate (GTP). GTP binding induces conformational changes within the Gα subunit that lead the Gβγ to dissociate, so that both subunits are free to activate downstream effector proteins, thereby initiating a signalling cascade (27).

**Figure 1:**
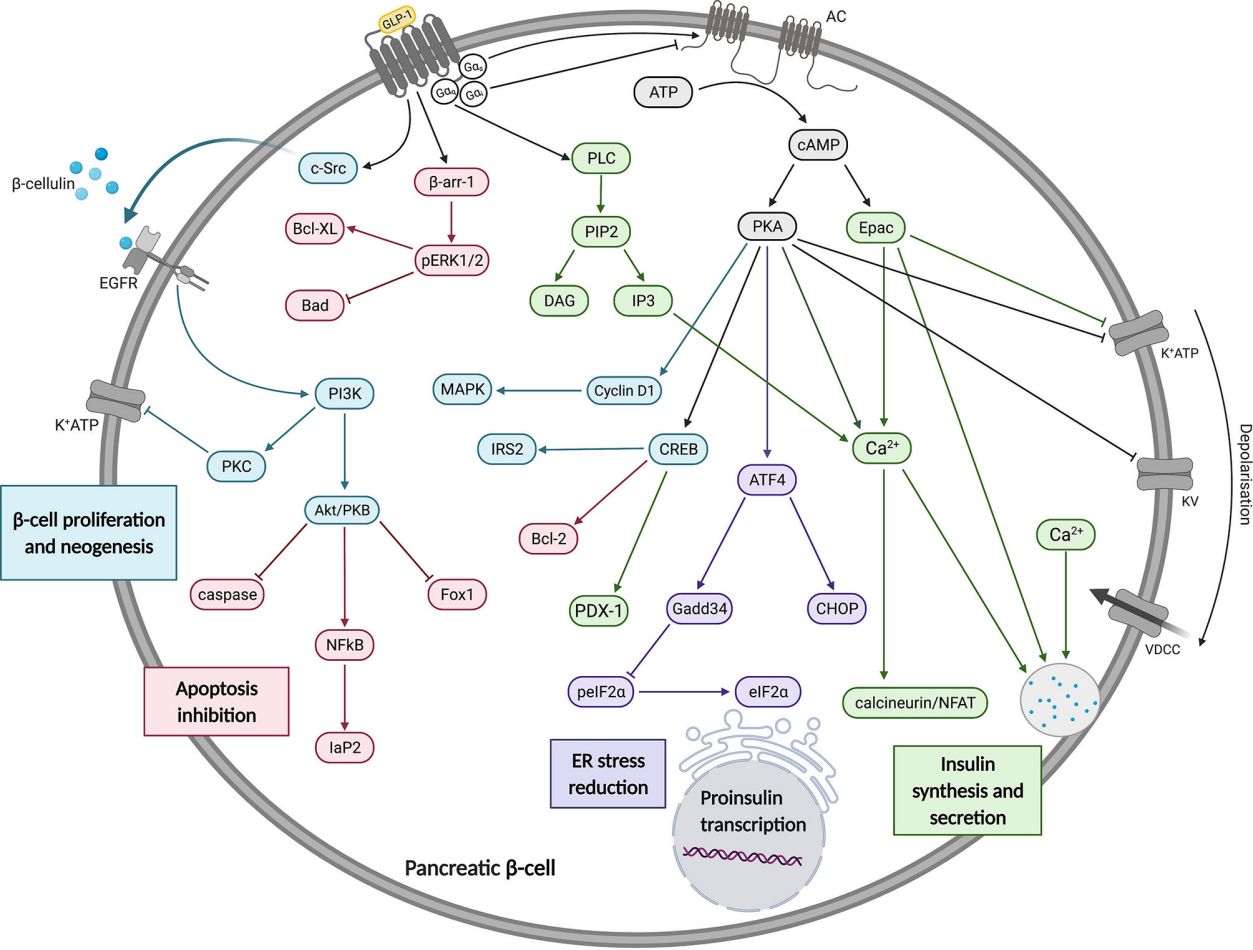
A summary of the effects of GLP-1 on insulin synthesis, secretion, beta cell proliferation, neogenesis and apoptosis inhibition. In pancreatic beta cells, GLP-1R signalling is predominantly *via* Gα_s_ which mediates increases in cAMP to activate Epac and PKA. These have a range of effects, as described in the main text and depicted in this figure. These and other pathways promote increases in insulin gene transcription, synthesis and secretion. Figure created using BioRender.com.

The GLP-1R is well known to couple with the Gα_s_ subunit, resulting in adenylate cyclase (AC) activation and cyclic adenosine monophosphate (cAMP) production (**Figure 1**). GLP-1R-stimulated pathways in pancreatic beta cells act within seconds to increase cAMP levels and minutes to potentiate GSIS. A rapid increase in cAMP is accompanied by activation of exchange protein activated by cAMP-2 (Epac2) and protein kinase A (PKA) (28). Activation of Epac2 reduces the concentration of ATP required to achieve closure of K_ATP_ channels promoting membrane depolarisation, Ca^2+^ influx and subsequent Ca^2+^-induced Ca^2+^ release from intracellular stores, insulin priming and finally insulin granule exocytosis (29–31). Activated PKA also promotes membrane depolarisation by directly phosphorylating the sulfonylurea receptor 1 (SUR1) and a regulatory subunit of K_ATP_ channels (32).

Additionally, PKA-mediated activation of cyclin D1 and MAPK was found to be crucial in the G1/S phase transition during the cell cycle, promoting beta cell neogenesis (33). GLP-1R signalling also attenuates the development of ER stress in beta cells through cAMP-dependent potentiation of Activating Transcription Factor 4 (ATF4) translation (34). These findings further support the claim that sustained GLP-1R agonism may result in disease modifying activity in people with T2D through enhancement or preservation of functional beta cell mass (35). GLP-1R also activates cAMP response binding element (CREB) which stimulates the expression of insulin transcription factor pancreatic and duodenal hombox gene-1 (PDX-1) in a cAMP/PKA-dependent manner (36). CREB was also shown to stimulate Insulin Receptor Substrate 2 (IRS2) gene expression which is essential for beta cell growth and survival in isolated beta cells and rodent models (37).

### Non-Gα_s_ Signalling Events in Beta Cells

GLP-1R is able to couple not only to Gα_s_, but also to other Gα subtypes including Gα_i_ and Gα_q_. The former acts in opposition to Gα_s_, leading to adenylate cyclase inhibition and reduced cAMP production. Whilst Gα_i_ coupling has been detected in response to GLP-1R activation (25, 38), it is not clear whether this is a significant factor in beta cells or an artefact of overexpression systems. There is more evidence that GLP-1R can couple to Gα_q_ in beta cells, as demonstrated by inositol triphosphate (IP_3_) turnover studies (39), diacylglycerol (DAG) production (40) and most convincingly, a Gα_q_ FRET biosensor (41). The latter study is interesting as it reported that the effects of GLP-1 on GSIS were predominantly Gα_s_-dependent under normoglycaemic conditions, but chronic beta cell depolarisation due to sulphonylurea treatment or hyperglycaemia led to an apparent switch in coupling preference to Gα_q_. Activation of Gα_q_ leads to phospholipase C (PLC) activation, allowing phosphatidylinositol 4,5-bisphosphate (PIP_2_) cleavage to generate DAG and IP_3_, with resultant effects on protein kinase C (PKC) activation and Ca^2+^ release from intracellular stores *via* IP_3_ receptor activation. Specifically, the putative Gα_q_-mediated mechanism is thought to involve PKC-mediated activation of an inward depolarising current carried by TRPM4/5 channels (42). The studies are not totally conclusive as cAMP can also activate PLC leading to intracellular Ca^2+^ increases (43).

Numerous reports show that GPCRs also induce distinct cellular responses *via* separate, non-G protein-dependent signalling cascades facilitated by the recruitment of β-arrestins, which act as scaffolds to promote interaction with and activation of mitogen-activated protein kinases (MAPKs) such as extracellular regulated kinase 1/2 (ERK1/2). Interestingly, a study using HEK293 cells with total elimination of all Gα subunits by a combination of CRISPR/Cas9 deletion and pharmacological inhibition suggested that, in fact, G proteins are essential for ERK1/2 phosphorylation and β-arrestins are not (44). However, it was also suggested that these results may have been artefacts of clonal selection rather than a true representation of the relative importance of G proteins and β-arrestins in this process (45). For the GLP-1R, β-arrestin-1 knockdown reduces ERK1/2 signalling in INS-1 cells, identifying a potential G protein-independent signalling axis required for GLP-1R-induced insulin secretion and coupling to protection against apoptosis (46, 47). In a study using INS-1 cells, GLP-1 facilitated β-cellulin release from the plasma membrane *via* β-arrestin-1-mediated recruitment of c-Src, leading to transactivation of the epidermal growth factor receptor (EGFR), a β-cellulin cognate receptor. This, in turn, sequentially activated phosphoinositide 3-kinase (PI3K) and downstream effectors protein kinase B (PKB/Akt), p38 MAPK and PKC contributing to DNA synthesis, gene expression, insulin synthesis and reduced apoptosis (48). Furthermore, mediated *via* PKB and nuclear factor-κB (NF-κB), GLP-1 also exhibits protective effects on beta cell glucotoxicity, lipotoxicity, glucolipotoxicity and stimulates transcription of the antiapoptotic genes *Iap-2* and *Bcl-2* (49). On the other hand, mice with beta cell-specific knockout of β-arrestin-1 or -2 did not show any defect in acute insulin secretory response to GLP-1RAs or alteration to beta cell mass (50, 51), although this was not investigated in detail for β-arrestin-2.

### Biased GLP-1R Agonism

The pleiotropic interactions between GPCRs and multiple signalling proteins has led to an important concept, particularly relevant to drug discovery, that efficacy has a quality as well as a magnitude. This concept, referred to as “biased agonism” or “signal bias”, is defined by ligands of the same receptor that show favoured and distinct coupling preferences to particular cellular effectors or pathways (7). Although detailed molecular mechanisms underpinning biased signalling are not yet completely understood, it is generally believed that biased ligands preferentially stabilise particular GPCR conformations for selective engagement with specific signalling pathways (52). One of the first reports in which the apparent biased behaviour of ligands was explicitly recognised showed that [Sar1,Ile4,Ile8]-AngII was unable to activate G protein signalling but still triggered β-arrestin recruitment and ERK1/2 phosphorylation at the angiotensin 2 type 1 receptor (53). In fact, further examples of apparently biased ligands can be found in the historical literature, pre-dating the emergence of signal bias as a fashionable pharmacological concept. One notable example relevant to class B GPCR pharmacology is desHis1,glu9-glucagon, a glucagon receptor “antagonist” which was found to stimulate IP_3_ production without cAMP generation (54).

Whilst the majority of currently approved therapeutic GLP-1RAs show balanced agonism between most pathways measured (55), an increasing number of biased GLP-1RAs have now been described in the preclinical literature. In line with major advances in the understanding of the structural basis of GLP-1R activation (24), the peptide N-terminus has emerged as an important determinant of biased agonism, with several studies reporting that β-arrestin recruitment can be selectively diminished by amino acid sequence substitutions and novel chemical entities in this region (56–60). Despite the putative role of β-arrestins as GLP-1R effectors coupled to both insulin release and protection against beta cell apoptosis (46, 47), most biased GLP-1RA studies have shown that reduced β-arrestin recruitment is associated with enhanced insulin secretion, at least under conditions of sustained stimulation. This is presumed to be a consequence of β-arrestin-mediated desensitisation and/or downregulation of the GLP-1R (61). Studies in rodent models of T2D show improved glucose tolerance with G protein-biased compared to balanced GLP-1RA treatments, with these benefits progressively apparent later in the dosing period, consistent with a desensitisation-related mechanism. Recently, tirzepatide, a clinical candidate dual incretin receptor agonist that targets both the GLP-1R and the closely related glucose-dependent insulinotropic polypeptide receptor (GIPR), was found to show profound biased agonism characterised by minimal β-arrestin recruitment at the GLP-1R (62, 63). Tirzepatide appears to be highly effective for T2D in human trials (64), although it is not yet possible to know how much the biased GLP-1R agonism contributes to this.

## Endocytic GLP-1R Trafficking

**Figure 2:**
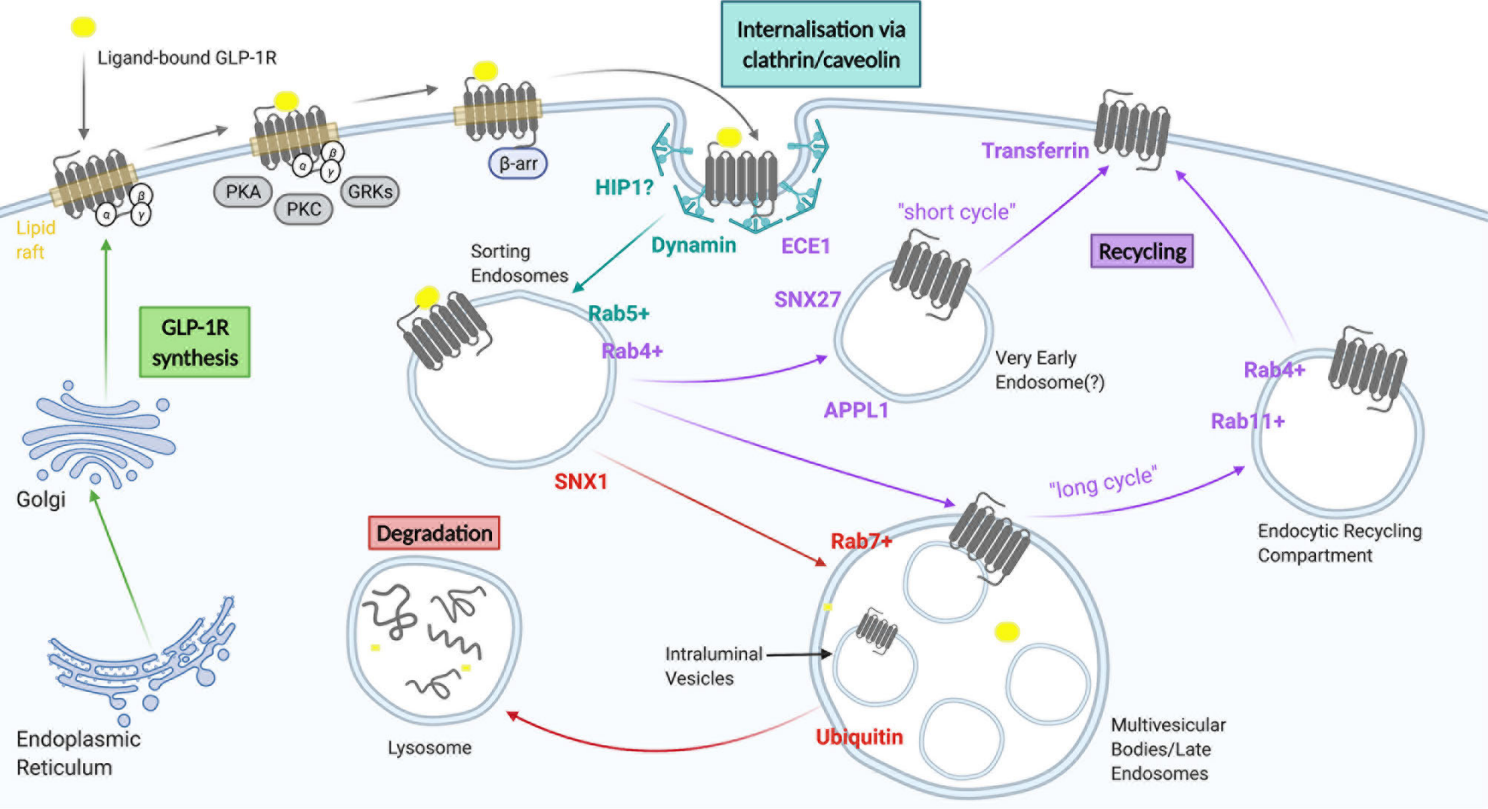
A summary of GLP-1R trafficking. GLP-1 receptors are internalised through clathrin-dependent and/or clathrin-independent mechanisms and subsequently transported to sorting endosomes/early endosomes. From here they can either be recycled back to the plasma membrane *via* a fast recycling pathway (short cycle), a slow recycling (long cycle), or be targeted for degradation to lysosomes *via* multi vesicular bodies/late endosomes. Figure created using BioRender.com.

Most ligand-activated receptors undergo endocytosis, a process recognised to serve a number of purposes, including regulation of cell surface receptor levels to modulate cellular responsiveness to continued ligand exposure, and alternative sorting of receptors towards one of the cell surface recycling pathways for sustained signalling, or towards lysosomal targeting for receptor degradation to achieve signal termination and long term desensitization (or down-regulation) (65). The endocytic pathway is composed of a continuum of intracellular vesicular compartments showing varying levels of maturation, including early or sorting endosomes, multivesicular or late endosomes, endocytic recycling compartments and lysosomes (66), which are in turn interconnected with a number of other intracellular organelles including the Golgi apparatus, the ER and mitochondria (67–70). Activated GLP-1R undergoes rapid endocytosis, with almost complete disappearance of surface receptors within 15 minutes of exposure to a maximal agonist concentration (55). Proposed molecular determinants of GLP-1R trafficking are summarised in **Figure 2** and discussed in more detail below.

### GLP-1R Endocytosis – Clathrin-Dependent or -Independent?

The GLP-1R has been previously reported to follow a dynamin-dependent, but either clathrin-mediated (CME) or clathrin-independent (CIE) endocytosis pathway (71–73), which represent the two main mechanisms underlying receptor endocytosis. Studies performed in CHO and CHL cells recombinantly expressing GLP-1R revealed internalisation *via* clathrin-coated pits (74). GLP-1R also appeared to internalise *via* clathrin-coated pits in pancreatic beta cells (71). This process, which followed clustering of immobilized receptors at membrane hotspots, involved cargo selection, clathrin-coat assembly, membrane bending, vesicle scission and uncoating (75). This canonical GPCR internalisation mechanism has been typically associated with β-arrestin recruitment, which can target GPCRs for internalisation *via* interaction with the β2 subunit of the AP2 clathrin adaptor, promoting movement of the GPCR-β-arrestin complex to vesicular pits where β-arrestin, AP2 and clathrin form a tripartite interaction (76, 77). However, it has also been observed that depletion of β-arrestins has a minimal impact on agonist-induced GLP-1R internalisation (55, 61, 71). The GLP-1R was found to possess an AP2-binding domain in its C-terminal tail allowing it to directly interact with AP2, minimising the need for additional intermediaries (78). The effects of β-arrestin-1 knockdown in INS-1 cells did not support a role for β-arrestin-1 in GLP-1R trafficking (46). Additionally, the endocytic accessory protein huntingtin-interactin protein 1 (HIP1) has been implicated in GLP-1R endocytosis (79); HIP-1 is a CME accessory protein that forms a bridging interaction between clathrin and AP2, thereby stimulating clathrin coated pit assembly (80).

In contrast to the above evidence supporting primarily a CME-dependent endocytosis pathway, experiments with small interfering RNA targeting clathrin did not appear to affect GLP-1R endocytosis in HEK293 cells, suggesting the existence of an alternative CIE-mediated mechanism (81). It is plausible that the GLP-1R can utilize more than one type of endocytosis mechanism, enabling it to deviate towards one of these alternative entry pathways in the event of blockade of its main pathway for internalization. While the nature of these alternative CIE pathways has not been elucidated, there have been some suggestions of a possible role for caveolin-1 in this process (72). GLP-1R contains a classic caveolin-1-binding motif within ICL2 enabling it to interact and colocalise with the protein intracellularly. Occurrence of this interaction was supported by using inhibitors of caveolin-1, with this mechanism of GLP-1R internalisation proposed to occur through activation of Gα_q_ proteins, followed by activation of PKC (69). It has also been suggested that β-arrestin-2 may play a role in GLP-1R trafficking mediated through caveolin-1 (46). Interestingly however, it has been reported that beta cell lines which display normal levels of GLP-1R endocytosis do not express caveolin-1, raising questions about the role of this protein in the control of GLP-1R internalisation (79). More generally, accumulating evidence within the general field of endocytosis has called into question whether a caveolin-mediated endocytic pathway exists at all (82), and the interaction between caveolin-1 and the GLP-1R might reflect alternative roles for this protein such as in the biosynthetic transport and targeting of mature receptors at the plasma membrane (60).

### The Role of Membrane Nanodomains in GLP-1R Endocytosis

The local organisation of GPCRs within plasma membrane cholesterol- and sphingolipid-enriched nanodomains (historically known as liquid-ordered domains or “lipid rafts”) can be dynamically regulated by ligand-induced activation, and is a key regulator of GPCR behaviours through compartmentalisation of both receptor and signalling effectors into membrane “hotspots” (83). Indeed, Gα_s_, the main G protein signalling subunit for the GLP-1R, was predominantly associated with cholesterol-rich detergent-resistant membrane fractions in beta cells (71). Moreover, adequate plasma membrane cholesterol levels were essential for efficient GLP-1R endocytosis *via* either clathrin-dependent and clathrin-independent pathways, as demonstrated by the virtual abolition of agonist-induced GLP-1R internalisation following cholesterol extraction with MβCD (methyl-β-cyclodextrin) in beta cells (71). Moreover, the GLP-1R undergoes agonist-dependent palmitoylation at its C-terminus, providing a mechanism for altered interaction and targeting of active receptors to these cholesterol-rich membrane regions (71). As well as a role in concentrating active GPCRs in particular membrane nanodomains enriched in signalling and trafficking proteins, direct interactions with cholesterol may alter the binding affinity of GPCRs (84, 85), suggesting that this lipid might allosterically modulate receptors by limiting their conformational flexibility, a possibility that remains to be investigated for the GLP-1R.

### Post-Endocytic GLP-1R Sorting/Trafficking

Post-endocytic GPCR trafficking occurs through a series of dynamically interconnected organelles crucial for the sorting of receptors to different intracellular compartments (86). The passage of GLP-1R through different beta cell endosomal compartments has been examined using electron microscopy (55, 87). Sorting endosomes, also known as early endosomes (EE), are the first organelles receiving the endocytosed receptor-containing vesicles, and are typically marked by Rab5 (88). The expression of a mutant Rab5 in beta cells inhibited internalisation of the GLP-1R, suggesting a crucial role for the sorting of receptors to Rab5-positive endosomes (89). From the limiting membrane of EEs, GPCRs may be recycled back to the plasma membrane *via* a “short/fast cycle” of trafficking from a Rab5-positive to a Rab4-positive endocytic compartment, *via* a “long/slow cycle” by entering Rab11-positive perinuclear recycling endosomes, or towards lysosomal degradation *via* retention in intraluminal vesicles (ILVs) of multivesicular bodies (MVBs), generated by maturation of EEs, a process that also causes termination of signalling, and subsequent trafficking to Rab7-positive late endosomes and lysosomes (90). High efficacy GLP-1RAs such as exendin-4 and GLP-1, when applied at high concentrations, target GLP-1Rs primarily towards retention in ILVs and lysosomal degradation (55, 79, 87). A retromer-associated factor, sorting nexin 1 (SNX1), was found to play a crucial role in targeting GLP-1R towards lysosomal degradation by restricting receptor recycling (79), but other molecular events governing this process are not well understood. For example, preliminary experiments have shown that receptor ubiquitination, a process typically involved in the canonical Endosomal Sorting Complex Required for Transport (ESCRT)-dependent targeting of endosomal proteins to ILVs within MVBs as a committing step towards lysosomal fusion, does not appear to apply to the GLP-1R, although alternative ESCRT-dependent but ubiquitin-independent, as well as ESCRT-independent mechanisms of sorting into ILVs have also been described, including for some GPCRs such as the protease-activated receptor PAR1 (91). Agonist-internalised GLP-1R colocalises with transferrin, a marker for recycling (92), and about 10 to 30% of internalised GLP-1R returns to the cell surface per hour after exendin-4 or GLP-1 treatment, respectively (55, 87). It has been shown in beta cells that another retromer-associated factor, sorting nexin 27 (SNX27), plays a role in cargo selection and GLP-1R sorting from early to recycling endosomes, with SNX27-knockdown significantly reducing GLP-1R recycling (79). This is due to its association with actin/sorting/nexin/retromer tubule (ASRT) and the Wiskott-Aldrich syndrome protein and SCAR homologue complex (WASH) (93). A “two-barcode” mechanism for GPCR recycling was hypothesised, acting through the endosomal ASRT pathway, involving both a PDZ-binding domain and phosphorylation of specific serine residues in the C-terminal domain (94). However, the GLP-1R does not possess a canonical PDZ-binding domain (79). Another aspect that requires further investigation is the possibility of GLP-1R undergoing retromer-dependent endosome-to-Golgi retrograde transport, as well as possible interactions with alternative retromer-like endosomal retrieval complexes involved in the control of receptor recycling such as the recently described retriever and commander complexes (95).

Once internalized, the acidic endosomal pH disrupts ligand-receptor interactions and influences post-endocytic GPCR trafficking, presumably as ligand-bound *versus* apo-state GPCR conformations show altered engagement, directly or indirectly, with local trafficking effectors. Accordingly, ligand binding affinity appears to be an important determinant of GLP-1R trafficking. High affinity agonists that remain bound to GLP-1R within the endosomal pathway tend to result in greater lysosomal targeting of the receptor, whereas lower affinity agonists preferentially lead to receptor recycling (87). As a further factor likely to influence ligand-receptor complex stability, the metalloprotease endothelin-converting enzyme-1 (ECE1) was also shown to colocalise with internalised GLP-1Rs, and presumed to facilitate GLP-1R recycling *via* proteolytic processing of endosomal GLP-1 (96), as previously shown for other GPCRs (97). Indeed, slow-recycling exendin-4 is highly resistant to ECE1, whereas GLP-1, which leads to faster GLP-1R recycling, is rapidly degraded by ECE1 at low pH (87). GLP-1RAs that carry a fatty acid chain to promote reversible binding to albumin, e.g. liraglutide and semaglutide, participate indirectly in recycling of albumin *via* the neonatal Fc receptor (FcRn) (98); and whilst this is an important factor determining the long half-life of these GLP1-RAs in the circulation, it is not known whether this phenomenon is relevant to GLP-1R recycling in beta cells.

### Caveats to GLP-1R Trafficking Studies Reported in the Literature

It is important to note that many studies investigating GLP-1R trafficking have used immortalised cell lines overexpressing the GLP-1R, typically stimulated with high agonist concentrations that are well above the physiological range and may even exceed the likely peak concentrations of pharmacological GLP-1RAs *in vivo*. These experimental conditions are to a certain extent mandated by the limitations of available methods to monitor the redistribution of the endogenous, agonist-stimulated GLP-1R. For example, “feeding” of GLP-1R extracellular-domain-recognising antibodies to monitor endocytosis is precluded as they compete for the orthosteric ligand binding site (99), and fluorescent agonist ligands dissociate from the receptor after entering the endocytic pathway so are informative only for the initial internalisation phase. Appending biorthogonal tags such as HALO- or SNAP-tags to label the endogenous GLP-1R genomic sequence could allow a better understanding of trafficking events without the use of exogenous expression, but has not been reported to date. The use of high ligand concentrations leads to large, easy to measure changes in GLP-1R redistribution, but it is possible that mode of endocytosis could shift as receptor occupancy increases (100). More sophisticated imaging-based approaches to monitor individual endocytosis events e.g. using total internal reflection fluorescence microscopy (TIRFM) combined with single particle tracking (SPT) would allow these phenomena to be observed at physiological agonist concentrations. Additionally, adapting assays for longer stimulation times but with lower ligand concentrations would aid in understanding how GLP-1R agonist drugs influence GLP-1R trafficking over the many hours or days that they persist in the circulation.

## GPCR Trafficking in the Spatiotemporal Control of Intracellular Signalling

Once thought to be solely constrained to the plasma membrane, GPCR signalling is now known to originate from various intracellular locations (101–103). Endocytic trafficking alters the location and duration of certain receptor-mediated signalling pathways and is thus a crucial regulator of specific cellular responses (104, 105). The possibility of endosomal cAMP generation has attracted considerable interest as a potential means to achieve sustained intracellular responses (106). Whilst the broad categories of “plasma membrane signalling” *versus* “endosomal signalling” are now well established, it should be acknowledged that subclassification of signals at point source is possible, e.g. from liquid-ordered *versus* liquid-disordered plasma membrane nanodomains (71).

Endosomal signalling by the GLP-1R has now begun to be explored. Internalised GLP-1Rs were shown to participate in compartmentalised cAMP generation, contributing to insulin granule exocytosis in cultured pancreatic beta cells before being directed to lysosomes for degradation (107). A follow up study showed that Gα_s_ is recruited to activated GLP-1R-containing Rab5-positive EEs (89). Inhibition of GLP-1R internalisation using dominant negative dynamin constructs reduced GLP-1R cAMP responses (73, 108). Additionally, A-kinase anchoring proteins (AKAPs) target PKA to discrete subcellular locations of cAMP production, compartmentalising Ca^2+^ and cAMP signalling, a crucial mechanism for the regulation of insulin secretion (109, 110).

Notwithstanding the importance of signal compartmentalization and endosomal signalling, long term reduction in GPCR abundance at the cell surface as a result of sustained agonist-mediated endocytosis without sufficient compensatory recycling (i.e. downregulation) is a clear potential limiting factor in the capacity of a cell to generate prolonged signalling responses in the face of chronic ligand exposure. Compared to other class B GPCRs, including the glucagon and glucose-dependent insulinotropic polypeptide receptors (GCGR, GIPR), endocytosis of the GLP-1R is rapid and extensive (61, 73), which could quickly lead to depletion of plasma membrane receptors, particularly when combined with preferential targeting to lysosomal degradation, a situation in which the biosynthetic pathway might not be able to replenish the pool of membrane receptors with sufficient efficiency. GLP-1R endocytosis and subsequent endocytic trafficking therefore may both enhance and attenuate signalling, with numerous factors such as ligand efficacy, concentration and exposure period as well as receptor clustering and nanodomain segregation all potential determinants of which process dominates.

### The Importance of Receptor Trafficking in Biased GLP-1R Agonism

The endocytic trafficking effects of a number of biased GLP-1RAs have been reported (55, 57–59, 87, 111). Typically, G protein-biased peptides with reduced β-arrestin recruitment efficacy also show attenuations in GLP-1R internalisation propensity, along with faster recycling back to the plasma membrane and reduced lysosomal degradation. Despite the canonical role of β-arrestins in clathrin-mediated endocytosis, it does not appear that the deficient β-arrestin response of biased agonists is directly responsible for the their distinct trafficking profiles, as agonist-induced GLP-1R endocytosis is minimally affected by the total absence of both β-arrestin isoforms (55, 61, 71). Instead, the reduced β-arrestin recruitment of slow-internalising, fast-recycling agonists is rather considered as a bystander marker for this type of trafficking behaviour, whose main role is in preventing rapid receptor desensitisation. An important characteristic that appears to determine the propensity to induce GLP-1R internalisation is the binding affinity displayed by the different agonists, a process that is intimately linked with their capacity to trigger receptor clustering and nanodomain segregation prior to internalisation (71). Indeed, allosteric modulation of binding affinity can render a weakly internalising agonist fast internalising properties (71). Additionally, different intra-endosomal GLP-1R-ligand dissociation rates observed with different biased GLP-1RAs is one factor that influences the receptor post-endocytic targeting, with faster dissociating biased GLP-1RAs tending to lead to more receptor recycling (55, 87). However, the precise biophysical mechanisms and receptor-effector interactions that differ between different biased GLP-1RAs and explain their differential endocytic rates have not been fully elucidated.

The fact that GLP-1R endocytosis promotes endosomal cAMP signalling but is also ultimately a route to receptor downregulation means that, irrespective of the mechanisms by which biased GLP-1RAs achieve their altered trafficking responses, they are likely to contribute to their overall signalling profiles. Endosomal *versus* plasma membrane signalling with a panel of GLP-1RAs was compared using targeted FRET cAMP biosensors (pmEpac2 and cytoEpac2), revealing that exendin-4 is biased in favour of cytoplasmic cAMP production compared to liraglutide (108). Aside from this study, the endosomal signalling characteristics of more recently invented biased GLP-1RAs with profoundly altered trafficking profiles have not been reported. This is a key gap in the literature, as it is not yet established whether the steady state preservation of surface GLP-1Rs seen with some biased GLP-1RAs is the primary result of a failure to enter the endocytic pathway (which would preclude endosomal signalling), or the accelerated rapid recycling from early endosomal compartments (which may permit endosomal signalling), or a combination of both processes. Targeted biosensor studies to investigate this phenomenon are required to address this issue. It is known however that lipid nanodomain-specific cAMP generation, measured using the raft-anchored cAMP sensor ^T^Epac^VV^-Lyn (112), is selectively enhanced by the β-arrestin-biased GLP-1RA exendin-asp3 over the G protein-biased exendin-phe1, matching the relative propensity of these agonists to stimulate segregation of GLP-1R into lipid rafts (71).

Whilst their endosomal signalling characteristics are not yet known, it is well established that some of the biased GLP-1RAs with reduced tendency to promote GLP-1R internalisation do indeed cause less beta cell GLP-1R degradation over prolonged periods of stimulation (55, 87). As long periods of receptor-drug exposure are maintained with therapeutic GLP-1RAs that have extended pharmacokinetic stability, these studies provide useful information on the likely differential impact of biased GLP-1RAs on target downregulation. Whilst this phenomenon has not yet been studied *in vivo*, various reports demonstrate that the net result is that poorly internalising GLP-1RAs preserve the responsiveness of pancreatic beta cells, as measured by cumulative insulin secretion *in vitro* over many hours and by rechallenge experiments to reveal different rates of homologous desensitisation (55, 87), and hence have a beneficial net effect despite potential losses of intracellular signalling. The implication of these findings is that, even if endosomal signalling is an important component of GLP-1R agonism, the “benefits” of increased steady state plasma membrane receptors with certain biased GLP-1RAs might exceed the potential reductions in endosomal signalling that might accompany this phenomenon.

## Conclusions

The GLP-1R plays a pivotal role in glucose homeostasis, insulin synthesis and potentiation of GSIS and has accordingly become a major therapeutic avenue for the treatment of T2D. It is apparent that the GLP-1R signalling-trafficking system is highly complex, comprising of many steps and can provide a platform for exquisite control of signalling events. The development of biased GLP-1RAs has provided a suite of tools for the reprogramming of GLP-1R endocytosis and post-endocytic sorting and a means to achieve distinct signalling patterns at different subcellular locations. The physiological and therapeutic implications of these processes are only now beginning to be explored.

Substantial progress has been made in our understanding of GLP-1R signalling and trafficking, such as the perpetuation and termination of signalling within endosomal compartments. However, significant gaps still remain to be resolved. The molecular mechanisms governing GLP-1R endocytosis are yet to be clearly defined, with a major role for typical candidates such as β-arrestins being, surprisingly, not supported by most of the evidence. Whilst the differences in post-endocytic GLP-1R sorting and trafficking with different GLP-1R ligands has been observed and described using an expanding number of imaging techniques, a comprehensive mechanistic explanation of these processes is still lacking. Moreover, the majority of studies have been performed in heterologous or insulinoma cell lines, often at high ligand concentrations, and confirmation in primary beta cells and *in vivo* will ultimately be required to determine the physiological importance of these processes. Finally, translating the study of the mechanisms governing GLP-1R trafficking to other cellular systems beyond the pancreas represents a further frontier in our understanding of the importance of these processes in GLP-1R biology. For example, it is postulated that GLP-1R-mediated endocytic carriage across tanycytes or endothelial cells is a specific mechanism by which GLP-1RAs access the brain (55, 113–115), although non-receptor-mediated mechanisms such as adsorptive transcytosis are also suggested to be important (116).

Overall, the promising results observed using biased GLP-1RAs with distinct trafficking profiles in preclinical studies have raised the possibility that this is a therapeutically viable strategy to confer greater efficacy and/or tolerability for the treatment of T2D. Dedicated studies in humans are now required to test this hypothesis.

## Author Contributions

AM, AT, and BJ wrote the manuscript. All authors contributed to the article and approved the submitted version.

## Funding

The Section of Endocrinology and Investigative Medicine is funded by grants from the MRC, BBSRC, NIHR, and is supported by the NIHR Biomedical Research Centre Funding Scheme. The views expressed are those of the authors and not necessarily those of the funders. AT acknowledges funding from Diabetes UK. BJ acknowledges support from the Academy of Medical Sciences, Society for Endocrinology, The British Society for Neuroendocrinology, the European Federation for the Study of Diabetes, and an EPSRC capital award. BJ and AT also received funding from the MRC (MR/R010676/1) and the European Federation for the Study of Diabetes. The authors declare having received separate funding from Sun Pharmaceuticals. This funder had no involvement in the present study.

## Conflict of Interest

The authors declare that the research was conducted in the absence of any commercial or financial relationships that could be construed as a potential conflict of interest.
